# Outrunning free radicals in room-temperature macromolecular crystallography

**DOI:** 10.1107/S0907444912012553

**Published:** 2012-06-15

**Authors:** Robin L. Owen, Danny Axford, Joanne E. Nettleship, Raymond J. Owens, James I. Robinson, Ann W. Morgan, Andrew S. Doré, Guillaume Lebon, Christopher G. Tate, Elizabeth E. Fry, Jingshan Ren, David I. Stuart, Gwyndaf Evans

**Affiliations:** a Diamond Light Source, Harwell Science and Innovation Campus, Didcot OX11 0DE, England; b OPPF-UK, Research Complex at Harwell, Rutherford Appleton Laboratory, Didcot OX11 0FA, England; c Division of Structural Biology, The Henry Wellcome Building for Genomic Medicine, Roosevelt Drive, Oxford OX3 7BN, England; d NIHR–Leeds Musculoskeletal Biomedical Research Unit and Leeds Institute of Molecular Medicine, University of Leeds, Leeds LS9 7FT, England; e Heptares Therapeutics Ltd, BioPark, Welwyn Garden City AL7 3AX, England; f MRC Laboratory of Molecular Biology, Hills Road, Cambridge CB2 0QH, England

**Keywords:** radiation damage, room temperature, dose rate, free radicals

## Abstract

A significant increase in the lifetime of room-temperature macromolecular crystals is reported through the use of a high-brilliance X-ray beam, reduced exposure times and a fast-readout detector. This is attributed to the ability to collect diffraction data before hydroxyl radicals can propagate through the crystal, fatally disrupting the lattice. Hydroxyl radicals are shown to be trapped in amorphous solutions at 100 K. The trend in crystal lifetime was observed in crystals of a soluble protein (immunoglobulin γ Fc receptor IIIa), a virus (bovine enterovirus serotype 2) and a membrane protein (human A_2A_ adenosine G-protein coupled receptor). The observation of a similar effect in all three systems provides clear evidence for a common optimal strategy for room-temperature data collection and will inform the design of future synchrotron beamlines and detectors for macro­molecular crystallography.

## Abstract 2

A systematic increase in lifetime is observed in room-temperature protein and virus crystals through the use of reduced exposure times and a fast detector.

## 1. Introduction   

The cryocooling of crystals in macromolecular crystallography (MX) greatly increases their lifetime in the X-ray beam, and for this reason the vast majority of synchrotron-based MX is performed using crystals held at 100 K in an open-flow nitrogen cryostat (Garman & Owen, 2007 ▸). Despite the advantages provided by cryocooling, there is still considerable interest in carrying out room-temperature (RT) crystallo­graphy at synchrotron sources, and this has been reflected by the development of dedicated sample environments at several facilities (Jacquamet *et al.*, 2004 ▸; Bingel-Erlenmeyer *et al.*, 2011 ▸; Axford *et al.*, 2012 ▸). The reasons for this interest are twofold. Firstly, screening of crystals *in situ* removes the invasive and potentially destructive step of crystal mounting and eliminates confounding factors such as cryoprotection and crystal handling when establishing optimal crystallization conditions. Secondly, some macromolecules, in particular viruses, prove to be difficult or indeed impossible to successfully cryocool, precluding data collection at 100 K. Additional motivation for collecting data at room temperature comes from recent work suggesting that data collection at 100 K can hide conformational diversity (Fraser *et al.*, 2011 ▸). In both cases radiation damage becomes a limiting factor during data collection and it is often impossible to collect a complete data set from a single crystal, or indeed a small number of crystals.

Several systematic radiation-damage studies at 100 K have shown no, or little, dose-rate effect (Garman, 2010 ▸). Accrued damage is only a function of the dose absorbed by the sample, and not of the manner in which it is deposited. In contrast, RT studies using both rotating-anode and synchrotron sources have shown a significant variation in crystal lifetime as a function of the rate at which dose is deposited in the crystal (Blake & Phillips, 1962 ▸; Southworth-Davies *et al.*, 2008 ▸; Rajendran *et al.*, 2011 ▸; Warkentin *et al.*, 2011 ▸). A dose-rate effect has also recently been observed at 260 K (Warkentin *et al.*, 2012 ▸). These observations are consistent with data-collection times being comparable with the timescales of X-­ray-induced chemical reactions and raise the exciting possibility of significantly increasing the amount of data that can be collected from a crystal by changing the way in which data are collected. The work reported here aims to address two questions that have yet to be answered fully. Firstly, what dose-rate effects exist when RT macromolecular crystals are exposed to the very high dose rates (∼1 MGy s^−1^) accessible at undulator beamlines and, secondly, on what timescales do dose-rate effects become significant?

Recent advances in detector technology have resulted in a paradigm change in the way data can be collected at MX beamlines. Until recently, the *de facto* standard was a CCD capable of reading out a single frame every 1–2 s, although this maximum frame rate is not realised at many beamlines owing to the need to ready the goniometer between images. We refer to this method of data collection as ‘stop–start’. The advent in MX of large-area pixel-array detectors (PADs; Eikenberry *et al.*, 2003 ▸) and, more recently, fast CCDs capable of reading out up to 30 frames per second (fps) dramatically changes the timescales on which it is possible to collect diffraction data, allowing so-called shutterless, or continuous, data collection. Continuous data collection truly opens a new temporal dimension in crystallography, potentially allowing the collection of diffraction data before damage can accumulate in, or propagate through, the sample. Damage has been observed to progress during the time following exposure to X-rays when the X-ray shutter is closed (Blundell & Johnson, 1976 ▸; Warkentin *et al.*, 2011 ▸): the absence of a pause during data collection eliminates this dark progression of damage, potentially allowing the collection of more data from a given sample. Continuous data collection enables for the first time ready investigation of the diffractive properties of protein crystals on subsecond timescales. The combination at I24, Diamond Light Source of a high-brilliance beamline providing ∼10^12^ photons s^−1^ into ∼10 × 10 µm and a fast-readout detector provides the opportunity to investigate RT radiation damage in weakly diffracting macromolecular crystals on short timescales.

In the work described here, the X-ray-induced decay of protein and virus crystals has been monitored as a function of dose rate and detector-readout mode in order to establish whether increased lifetimes result (i) when diffraction data are collected continuously without repeated pauses in X-ray irradiation while the detector reads out and (ii) when data are collected with increased incident fluxes for a shorter period of time, breaking the linear relationship between absorbed dose and accrued damage observed at cryogenic temperatures. The identity of the free radicals responsible for the difference in the radiation susceptibility of macromolecular crystals has been probed using UV–Vis absorption spectroscopy.

## 2. Methods   

### 2.1. Crystallization   

Immunoglobulin γ Fc receptor IIIa (FcγRIIIa) crystals were grown as described previously (Axford *et al.*, 2012 ▸) and were loop-mounted within a capillary during data collection. Crystals of 20–30 µm in size grew in space group *P*6_1_22, with unit-cell parameters *a* = *b* ≃ 60.6, *c* ≃ 214 Å, in 0.5 *M* NaCl, 0.1 *M* sodium citrate tribasic dyhydrate, 2%(*v*/*v*) ethylene imine polymer pH 5.5. Bovine enterovirus serotype 2 (BEV 2) crystals of ∼30 µm in size were grown in space group *F*23, with unit-cell parameters *a* = *b* = *c* ≃ 436.6 Å, in 1.5 *M* ammonium sulfate, 0.1 *M* bis-tris propane pH 7.0 using the sitting-drop method (Walter *et al.*, 2005 ▸). Diffraction data were collected directly from crystals within the crystallization tray using the *in situ* setup available at I24. Crystals of the A_2A_ adenosine G-protein coupled receptor (A_2A_AR) were grown in space group *C*2, with unit-cell parameters *a* = 76.5, *b* = 98.9, *c* = 79.5 Å, in 0.05 *M* Tris–HCl pH 7.6, 9.6% PEG 200, 22.9% PEG 300 as described by Lebon *et al.* (2011 ▸) and were loop-mounted within a capillary for data collection.

### 2.2. Diffraction data collection   

Diffraction data were collected on the microfocus beamline I24 at Diamond Light Source. The beam was defocused to 20 × 20 µm at the sample position to match the typical crystal size, with the focal plane of the beam shifted towards the detector by 40 mm. Data were collected at X-ray energies of 12.68 keV (FcγRIIIa crystals) and 12.8 keV (BEV 2 and A_2A_AR crystals). Diffraction data were recorded using a Pilatus 6M PAD (Dectris) modified to run at frame rates of up to 25 Hz. Diffraction data were collected in two experimental modes: ‘stop–start’ and ‘continuous’. During stop–start data collection the experimental shutter was closed for a 4, 8 or 12 s period between consecutive images, emulating a traditional MX experiment in which the detector takes a few seconds to read out and the hardware must be positioned in preparation for the next image. Owing to software and hardware limitations at the time of the experiments, a pause of less than 4 s was not possible. During continuous data collection the X-ray shutter remained open for the duration of the experiment, which is the default mode of operation for PADs on the Diamond MX beamlines.

X-ray diffraction data were analysed using *LABELIT* (Sauter & Poon, 2010 ▸). Using *DISTL*, the integrated signal strength given in pixel ADC units above the local background of all Bragg candidates was calculated on a per-image basis. This was defined as the diffracting power of the crystal, allowing rapid determination of the diffracting power as a function of image number. The dose in MGy absorbed by each crystal during a data set was determined using *RADDOSE* (Paithankar & Garman, 2010 ▸). Dose calculations showed that the crystals of FcγRIIIa, BEV 2 and A_2A_AR were subject to maximum dose rates of 0.69, 0.89 and 1.00 MGy s^−1^, respectively. In order to establish whether a dose-rate effect existed, data were collected with a range of exposure times from 0.04 to 4.09 s, with the beam being attenuated such that the dose absorbed per image was constant, *i.e.* no attenuation was used for exposure times of 0.04 s, while the beam was attenuated by 98.5% for 4.09 s exposures. All intensity decays were fitted with an exponential function of the form *I* = *a* + *b*exp(dose/*d*_*e*_). The quantity *d*_*e*_ represents the dose required for the diffracting power of the crystal to fall by 1/*e* and was taken as the crystal lifetime. To compare directly with other studies, in particular those carried out at 100 K, where a linear intensity decay is observed and the dose required for the diffracting power to fall by half is quoted, the lifetimes here should be multiplied by 2/*e*.

The Pilatus 6M is a paralyzable detector: for a dead-time period after each photon is recorded a detector pixel is ‘blind’ to incident X-rays. A correction is applied to the counts recorded by the detector to account for this. The combination of high fluxes and short exposure times used in these experiments mean that a count-rate correction may be significant for some reflections and, in the limit, would result in systematic underestimation of intensities. Appendix *A* [App appa] and Supplementary Fig. S1^1^ show that even for the most intense reflections observed from FcγRIIIa, A_2A_AR and BEV 2 the dose-rate correction is small and does not invalidate the model used.

### 2.3. UV–Vis data collection   

*In situ* UV–Vis absorption spectra were collected on beamline I02 at Diamond Light Source. Spectra were collected using mirror lenses (Bruker) mounted in an off-axis geometry and a deuterium halogen light source (Ocean Optics). Spectra were recorded over the wavelength range of 200–750 nm using a Shamrock 303 imaging spectrograph (Andor). Data were collected using an X-ray energy of 12.658 keV, an X-ray beamsize of 70 × 110 µm and a UV–Vis focal spot 100 µm in diameter. Spectra were collected using an exposure time of 40 ms with eight accumulations per spectrum written to disk.

## 3. Results   

The high doses required to record useful diffraction from the samples used in this study resulted in extremely rapid crystal decay. Crystal lifetimes can therefore only be determined over a small number of images (<25), resulting in some apparent variation in lifetime between crystals subjected to the same incident flux. To address this, and in order that overall trends in lifetime as a function of dose rate can be determined without crystal-to-crystal variation being a dominant factor, a large number of crystals have been used in this study (>110). All crystals exhibited an exponential decay in intensity and an exponential function with an *R* value greater than 0.9 could be fitted to the observed decay. It is important to note that the ability to record only a small number of images does not preclude structure solution: the structures of FcγRIIIa and BEV 2 have both been solved using RT data recorded *in situ* at I24 (Axford *et al.*, 2012 ▸).

**Figure 1:**
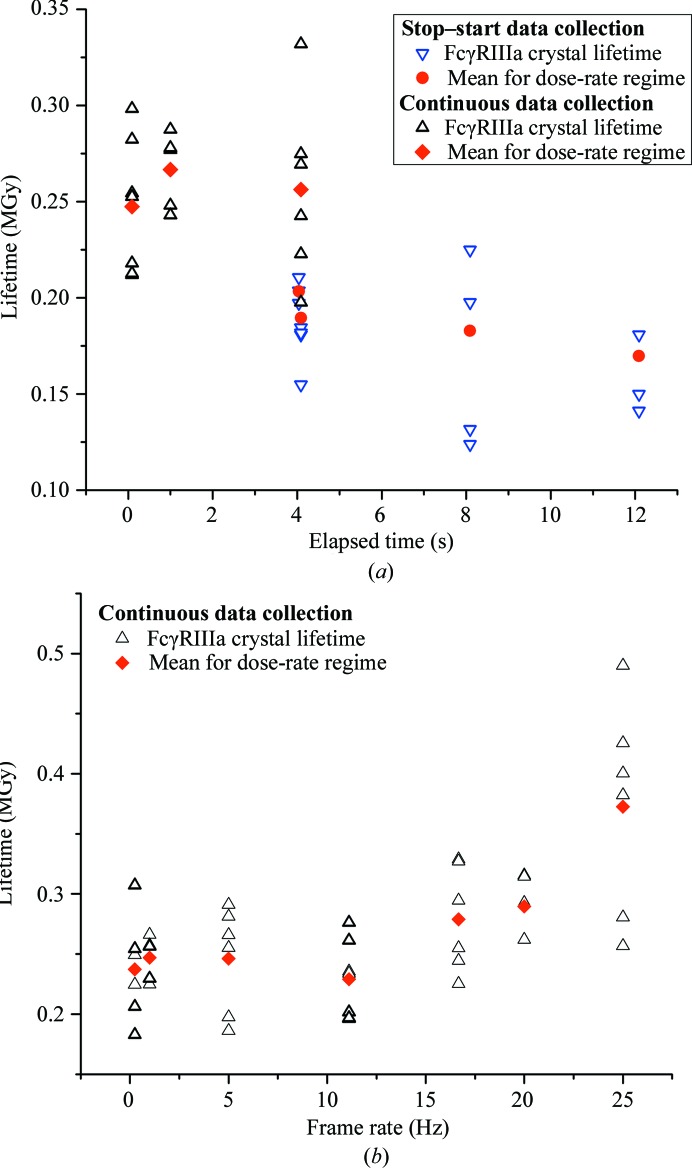
Comparison of FcγRIIIa crystal lifetimes in both stop–start and continuous data-collection modes. Data sets when the fast shutter was closed between images are termed ‘stop–start’ and are shown as blue open triangles, with the mean for each regime shown as a red circle. Data sets when the detector was read out continuously are shown as filled black triangles, with the mean shown as a red diamond (top). For all data, elapsed time is equal to the exposure time plus the shutter closed time between images (0 s in the case of continuous data collection). The bottom panel shows the lifetime of FcγRIIIa crystals as a function of frame rate. All data were collected in continuous data-collection mode, *i.e.* with the X-ray shutter left open for the duration of the experiment. Data for individual crystals are shown as black triangles and the means for each dose-rate regime are shown as red diamonds.

In order to investigate slow (timescales of ≥1 s) damage accumulation, diffraction data were collected from FcγRIIIa crystals in both stop–start and continuous data-collection modes. Continuous data were collected with exposure times per frame of 0.09 s (11 Hz), 1 s and 4.09 s (0.24 Hz), while stop–start data were collected with an exposure time of 0.09 s and a pause of 4, 8 or 12 s, emulating and exaggerating typical CCD shuttered data-collection modes used over the last 15 years at synchrotron beamlines. An additional stop–start data series was also collected with an exposure time of 0.04 s and a pause of 4 s. The incident beam was attenuated so that the absorbed dose per image was the same for each dose-rate regime. All data are summarized in Fig. 1 ▸(*a*). A systematically shorter crystal lifetime was observed for data collected using a stop–start approach (mean lifetime 0.186 MGy) in comparison to continuous data (mean lifetime 0.257 MGy). Within each data-collection mode there is very little variation in lifetime.

Shorter timescales were probed in FcγRIIIa crystals by collecting data continuously with exposure times of 4.09–0.04 s, corresponding to frame rates of 0.24–25 Hz (Fig. 1 ▸ *b*). Below 11 Hz (exposure times ≥ 0.09 s) the crystal lifetime is almost constant as a function of dose rate, but at higher frame rates (shorter exposure times) a systematic increase in lifetime becomes apparent. Below 11 Hz the mean lifetime is 0.257 MGy; when data are collected at 25 Hz (exposure time 40 ms) this increases to 0.373 MGy. This represents a significant increase on the mean lifetime of 0.186 MGy observed in the stop–start experiments.

**Figure 2:**
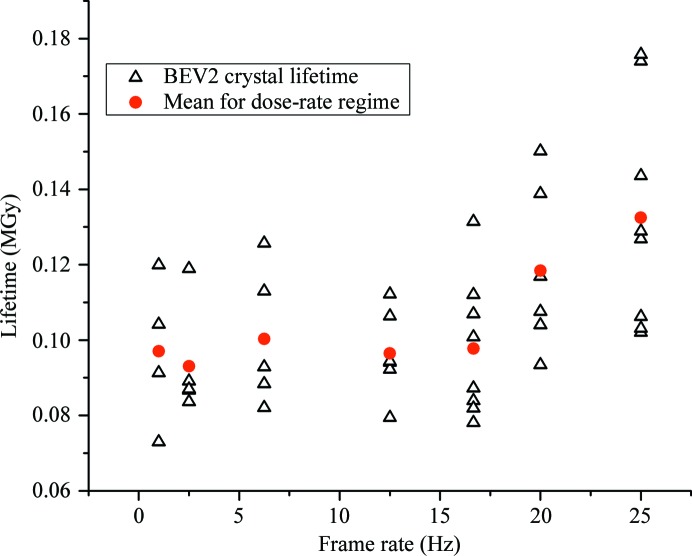
Plot showing variation in the lifetime of BEV crystals as a function of frame rate. At frame rates above 15 Hz an increase in the mean lifetime of the crystals is apparent. The dose per frame is kept constant for all crystals and frame rates.

The continuous exposure dose-rate experiments were repeated on BEV 2 crystals and a similar trend in crystal lifetime was observed (Fig. 2 ▸). In the case of BEV 2 the crystal lifetime was observed to be approximately constant up to frame rates of 16 Hz (exposure times ≥ 0.06 s), with a mean crystal lifetime of 0.097 MGy. At higher frame rates the mean lifetime increased, reaching 0.132 MGy at 25 Hz. The experimental approach for data collection from BEV 2 crystals differed in that data were collected from crystals in the crystallization drop rather than loop-mounted within a capillary. The methodology of the experiment was otherwise identical.

**Figure 3:**
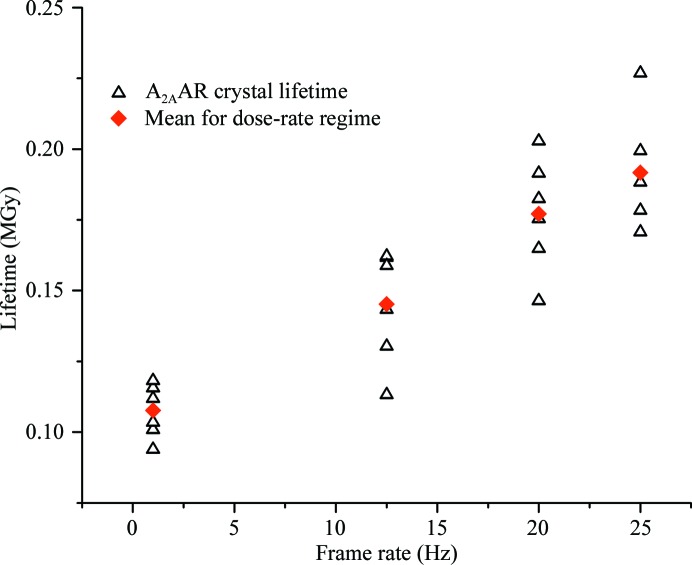
Plot showing variation in the lifetime of A_2A_AR crystals as a function of frame rate. A linear relationship between frame rate and lifetime can be observed. The dose per frame is kept constant for all crystals and frame rates.

Continuous exposure dose-rate experiments were also carried out on crystals of A_2A_AR. However, owing to the limited availability of crystals fewer dose-rate regimes could be probed. In contrast to FcγRIIIa and BEV 2, the lifetime of adenosine A_2A_AR crystals varied almost linearly as a function of dose rate, increasing from 0.108 MGy at 1 Hz to 0.192 MGy at 25 Hz (Fig. 3 ▸). While a clear increase in lifetime as a function of dose rate is apparent, the lack of dose-rate regimes probed make it impossible to establish whether a region with no dose-rate effect exists at low frame rates.

**Figure 4:**
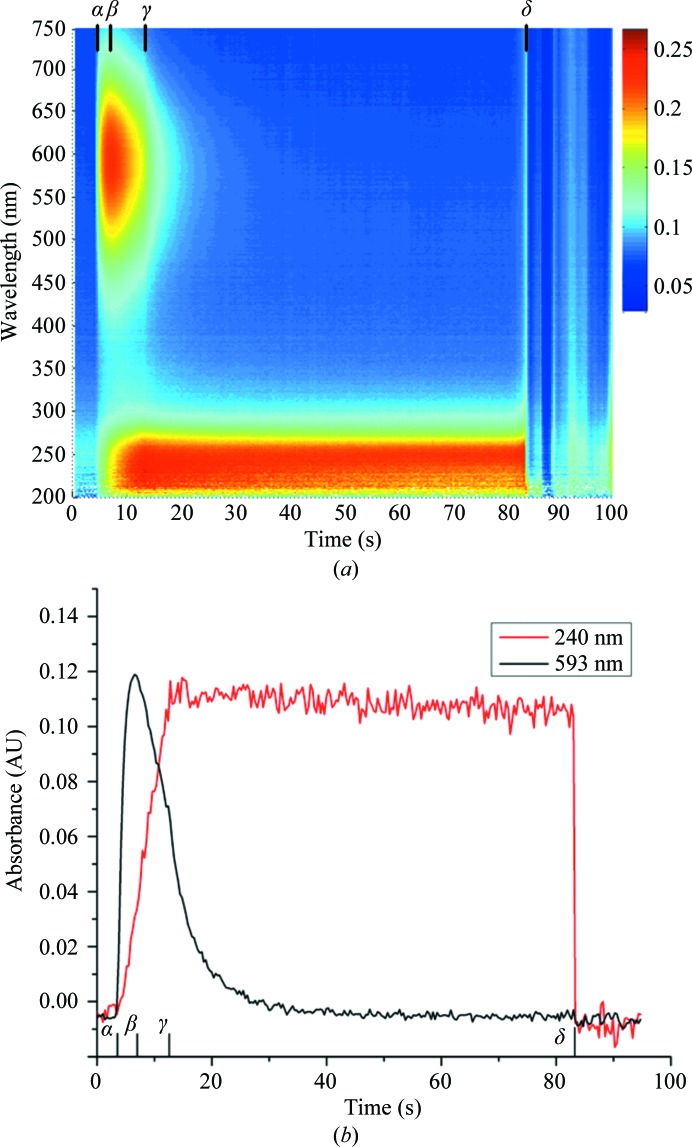
Change in the UV–Vis absorption spectra of FcγRIIIa crystallization solution at 100 K upon exposure to X-rays. (*a*) shows a contour plot showing changes at all wavelengths between 200 and 750 nm, while (*b*) shows the change in absorbance at 240 and 593 nm. The X-ray shutter is opened at α (*t* = 3.40 s), the 593 nm peak reaches a maximum at β (6.50 s), the X-ray shutter is closed at γ (12.40 s) and the 100 K nitrogen stream is blocked at δ (83 s).

**Figure 5:**
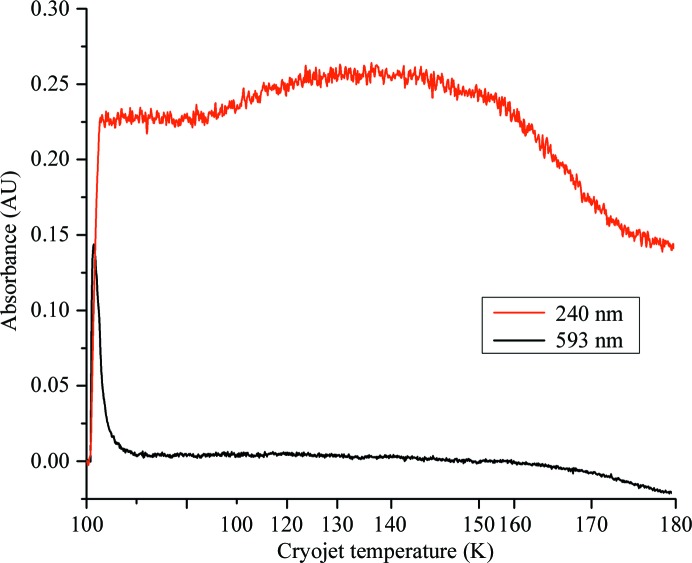
Change in UV–Vis absorbance of FcγRIIIa crystallization solution at 240 and 593 nm irradiated at 100 K and then warmed to 180 K. The *x* axis reflects the readback temperature from the cryojet controller; the temperature at the sample will differ from this. Note that this axis is nonlinear and approximate. At approximately 150 K absorbance at low wavelengths decreases significantly, indicating increased mobility of radicals. The absorbance at 240 K does not return to zero owing to discolouration and icing of the sample upon slow warming.

UV–Vis absorption data were collected from thin films of FcγRIIIa well solution held at 100 K. Before exposure to X-­rays the spectra showed no features and were flat over the wavelength range 200–750 nm (Fig. 4 ▸ *a*). Upon exposure to X-­rays a peak centred at 593 nm rapidly evolved and followed a first-order exponential decay (Fig. 4 ▸ *b*). A second peak at 240 nm grew at a slower rate and remained present until the 100 K nitrogen stream was blocked and the sample temperature was allowed to rise. The transient peak at 593 nm is indicative of the generation of aqueous, or solvated, electrons (McGeehan *et al.*, 2009 ▸), while we postulate that the peak at 240 nm is representative of the hydroxyl radical, as observed elsewhere (Hug, 1981 ▸; Janik *et al.*, 2007 ▸). Upon slower warming of FcγRIIIa well solution the absorbance at 240 nm was observed to remain approximately constant until ∼160 K (Fig. 5 ▸). Irradiation of thin films of sodium citrate alone, a principal component of the FcγRIIIa well solution, resulted in absorption peaks at 263 and 332 nm with no peak at 240 nm. UV–Vis absorption data were also collected from thin films of pure water, but the presence of microcrystalline ice made the collection of low-wavelength (<250 nm) data impossible. Nonetheless, a clear absorbance shoulder was observed to form at the low-wavelength limit upon exposure to X-rays, indicating the formation of hydroxyl radicals (Appendix *B* [App appb]). UV–Vis absorption data were also collected from thin films of FcγRIIIa well solution at RT: the spectra showed no change upon exposure to X-rays. A low-wavelength absorbance peak was also observed to form upon irradiation of solutions containing glycerol, but in this case an increase in absorbance at 240 nm is likely to indicate the breakdown of glycerol to malonic dialdehyde (Ivanova *et al.*, 2009 ▸).

**Figure 6:**
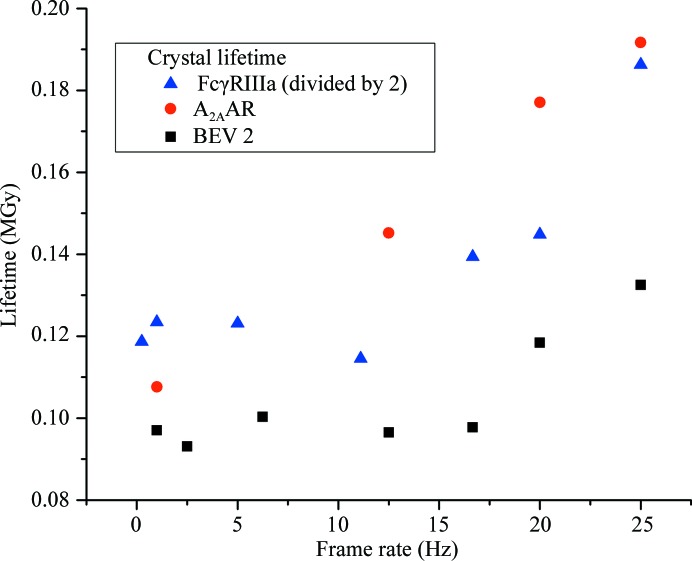
Lifetime of FcγRIIIa, BEV and A_2A_AR crystals as a function of dose. Note that the FcγRIIIa data have been scaled by a factor of two to facilitate comparison with the BEV 2 and A_2A_ data. All crystal types show an increase in lifetime at higher frame rates.

In order to compare the dose-rate effects in FcγRIIIa, BEV and adenosine A_2A_AR crystals, mean lifetimes are overlaid in Fig. 6 ▸. To facilitate comparison, the FcγRIIIa lifetimes have been divided by a factor of two, so that in all cases the crystal lifetime at 1 Hz is of the order of 0.1 MGy. The plot clearly shows a dose-rate effect in all systems studied, with increased crystal lifetimes observed when collecting data continuously at high frame rates (≥16 Hz).

## 4. Discussion   

This study provides clear evidence for a dose-rate effect in RT macromolecular crystallography when using the full flux of an undulator beamline at a third-generation synchrotron in conjunction with a fast-readout detector. This study extends previous work on RT data collection and radiation damage (Cherezov *et al.*, 2002 ▸; Southworth-Davies & Garman, 2007 ▸; Barker *et al.*, 2009 ▸; Rajendran *et al.*, 2011 ▸; Warkentin *et al.*, 2012 ▸). The FcγRIIIa, BEV 2 and A_2A_AR crystals used in this study were subjected to maximum dose rates of 689, 886 and 995 kGy s^−1^, which are comparable to the maximum previously reported (Warkentin *et al.*, 2012 ▸). In addition, the fast-readout detector enabled 25 fps to be collected. This is almost two orders of magnitude beyond previous studies with CCDs and image plates. At exposure times of less than 60 ms (>16 fps) lifetimes increase as a function of dose rate. We also found that the crystal lifetime reduced by a factor of two when there was a pause between frames within a data set when, for example, the X-ray detector is read out, in contrast to previous findings (Warkentin *et al.*, 2012 ▸). Despite differences in experiment design between this and other studies, it is interesting to note the similarity in the crystal lifetimes observed. RT lifetimes are of a similar magnitude, *i.e.* ∼0.2 MGy, and are approximately two orders of magnitude less than lifetimes at 100 K, suggesting that the processes that dominate room-temperature X-ray-induced damage are essentially complete on timescales shorter than even those studied here.

What are these processes? On very short timescales it has been demonstrated (using an X-ray free-electron laser with an X-ray pulse length of a few tens of femtoseconds) that very intense X-ray beams allow a massive X-ray dose to be deposited, leading to measurable diffraction from crystals at RT before the complete ionization of the structure destroys the lattice in less than 100 fs (Neutze *et al.*, 2000 ▸; Chapman *et al.*, 2011 ▸). In contrast, the processes in our experiments occur over timescales some ten orders of magnitude longer in crystals subjected to a much more modest dose rate.

At the X-ray energies used in macromolecular crystallo­graphy, photoelectric absorption accounts for most of the energy (∼84%) deposited in the crystal. Following such an interaction, atoms predominantly relax through ejection of an Auger electron, resulting in positive holes, and free electrons with high kinetic energy which are unlikely to return to the parent atom (Paithankar *et al.*, 2009 ▸). Owing to the high solvent content of macromolecular crystals^2^ much of the photoelectric absorption results in direct or indirect radiolysis of water, resulting in the formation of aqueous (solvated) electrons (e^−^_aq_) and hydroxyl radicals (·OH) (Klassen, 1987 ▸; Symons, 1999 ▸),
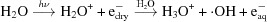
(Klassen, 1987 ▸). Approximate yields can be estimated through comparison with experiments carried out under anaerobic conditions (Sclavi *et al.*, 1997 ▸). For every 100 eV of energy absorbed under anaerobic conditions, the following numbers of radicals are obtained: 4.14 H_2_O, 2.7 H^+^, 2.7 e^−^_aq_ and 2.87 ·OH (Buxton, 1987 ▸). The mobility of radicals, and hence their ability to propagate through a unit cell and cause damage, changes significantly as a function of temperature. Hydrogen is mobile at temperatures above ∼30 K (Mao *et al.*, 2002 ▸). Interestingly, cooling protein crystals to below this temperature can have a deleterious effect. Trapped hydrogen results in a loss of short-range order, outweighing any gain resulting from a reduction in free-radical mobility (Meents *et al.*, 2010 ▸). Electrons and holes are mobile in proteins at temperatures down to 77 K (Jones *et al.*, 1987 ▸; Symons, 1995 ▸), suggesting that the large difference in the rates of damage at 100 K and RT does not arise from these species. In contrast, the mobility of ·OH is believed to change significantly between 100 K and RT. The ESR spectra of ice and poly(ethylene oxide) show ·OH to be trapped at 77 K (Brivati *et al.*, 1969 ▸; Zakurdaeva *et al.*, 2005 ▸) and upon warming to 115 K (Zakurdaeva *et al.*, 2005 ▸) to 130 K (Symons, 1999 ▸) ·OH becomes mobile and any spectroscopic signature disappears.

At 100 K aqueous electrons can move through the unit cell and rapidly react, with the result that their spectroscopic signature rapidly reaches a maximum (at ∼45 kGy; Owen *et al.*, 2011 ▸) and then decays (Fig. 4 ▸). Hydroxyl radicals remain trapped and are unable to react, so the characteristic ·OH band does not decay (Fig. 4 ▸). Upon warming of solutions to room temperature the ·OH band disappears in less than 0.3 s. At RT it was not possible to observe the formation of either aqueous electron or hydroxyl peaks, although the samples rapidly became discoloured. Upon controlled warming of FcγRIIIa solutions the ·OH band remained constant up to temperatures of ∼160 K. Above this temperature absorbance at 240 nm decreased, suggesting increased radical mobility, but did not return to zero. This may arise from discolouration of the sample, icing or the formation of further radical products which absorb at this wavelength. The change in mobility of ·OH between 100 K and room temperature indicated by these results together with previous studies suggest that ·OH radicals are the root cause of greatly increased rates of damage in RT crystallography.

For these experiments, samples were either loop-mounted within a capillary (FcγRIIIa and A_2A_AR) or held *in situ* within a crystallization tray (BEV 2) at 295 K for data collection. In the absence of active cooling by a gas stream it might be expected that X-ray-induced sample heating is significant and has an effect on crystal lifetime. Measurement of the temperature rise induced in a glass bead exposed to ∼3 × 10^12^ photons s^−1^ (energy 6.5 keV, beamsize 103 × 84 µm) in the absence of a gas stream revealed a temperature rise of ∼25 K over 5 s (Snell *et al.*, 2007 ▸). The observed temperature rise over 1 s was somewhat lower: ∼10 K. Taking the differing X-ray energy used, the significantly larger X-ray attenuation length and the smaller size of the protein crystals into account, it can be assumed that these observations provide an upper bound for the temperature rise in a protein crystal (Snell *et al.*, 2007 ▸). While large temperature rises may result from prolonged X-ray exposure, the above combined with the brief duration of the experiments described here means that temperature effects were not considered to be a critical factor determining crystal lifetime.

How do ·OH radicals cause damage? They are extremely reactive, with their rate of reaction being diffusion-limited (Dorfman & Adams, 1973 ▸; Swartz & Swartz, 1983 ▸; Xu & Chance, 2007 ▸). This reactivity is exploited in the field of footprinting, in which ·OH radicals are used to cleave nucleic acid or protein chains (Sclavi *et al.*, 1997 ▸; Xu & Chance, 2007 ▸). In proteins, a number of reaction pathways are possible: main-chain cleavage may occur either directly through attack at the α-carbon or proline oxidation or by radical transfer *via* side chains. Hydroxyl interaction *via* side chains is 10–10 000 times faster than with the backbone (Takamoto & Chance, 2006 ▸). The solvent composition has a significant effect on the efficiency of footprinting: buffers such as Tris, citrate, HEPES, MOPS, CAPS and CAPSO increase the lifetime owing to their un­favourable (in the case of footprinting) ·OH-quenching properties, whereas sodium cacodylate or phosphate buffers have been reported to have minimal quenching properties (Xu & Chance, 2007 ▸). Similarly, additives such as glycerol, ATP, EDTA and ADP have also been shown to significantly prolong the lifetime in footprinting experiments through quenching effects even at low concentrations: 1.0 m*M* glycerol can diminish the rate of radiolysis by a factor of five. It might therefore be expected that the lifetime of protein crystals varies significantly as a function of solvent composition. This has been demonstrated though the success of radical scavengers at room temperature in comparison to 100 K (Barker *et al.*, 2009 ▸; De la Mora *et al.*, 2011 ▸; Kmetko *et al.*, 2011 ▸). This is also reflected in the variation in the lifetime of protein crystals at RT as a function of both solvent composition and protein type, in contrast to 100 K where several studies determining crystal lifetime have shown remarkable agreement (Howells *et al.*, 2009 ▸). The increased lifetime of FcγRIIIa (*d*_e_ ≃ 0.24 MGy) crystals that we observe at low frame rates in comparison to BEV 2 (*d*_e_ ≃ 0.1 MGy) and adenosine A_2A_AR (*d*_e_ ≃ 0.11 MGy) may in part arise from the presence of citrate, a known ·OH quencher (Gupta *et al.*, 2007 ▸). None of the buffers characterized as efficient ·OH scavengers are present in the crystallization conditions of BEV 2 or A_2A_AR.

Figs. 1 ▸(*b*) and 2 ▸ show that for exposure times of >100 ms (<10 fps) the FcγRIIIa and BEV 2 lifetimes are independent of dose rate, suggesting that radical formation and diffusion are complete within these timescales (the limited availability of A_2A_AR crystals meant that only a single regime with an exposure time greater than 100 ms could be probed). Lifetime then increases with dose rate. In all of the systems studied the fact that the mean lifetime continually increases as a function of dose rate over a number of images of 100 ms or less is consistent with radical chemistry on timescales of this order.

There are three possible origins of a dose-rate effect on these timescales: self-recombination of radical species, the rate of diffusion of radicals through the solvent and quenching (radical absorption) within the bulk solvent.

Firstly, there may be significant radical recombination at high flux densities. In the experiments described here the primary yield of hydroxyl and other radicals is high: for every 100 eV absorbed 2.87 ·OH radicals are produced. Our maximum dose rate of ∼1 MGy s^−1^ results in the generation of approximately 1 × 10^5^ ·OH radicals, and a similar number of holes and aqueous electrons, per unit cell per second, or ∼4 × 10^3^ per image at 25 Hz. These radicals are not generated uniformly in the bulk solvent, but in regions a few nanometres in size called spurs (Hill & Smith, 1994 ▸). Recombination occurs when spurs overlap. It is therefore possible that on increasing the dose rate increased radical recombination occurs, reducing the number of radicals available to damage the protein lattice.

Secondly, radicals formed in the bulk solvent will take a finite time to diffuse in sufficient numbers to cause damage observable through diffraction. In this case, a lag phase reflects the time taken for the diffusion of ·OH through the bulk solvent. In line with the rate of diffusion being a limiting factor, the increase in atomic *B* factor has been observed to be greater close to solvent channels in thermolysin crystals at 160 K, with residue depth conferring a degree of protection from radiation damage (Juers & Weik, 2011 ▸).

Thirdly, radicals are quenched within the solvent. Dose-rate effects on timescales of less than 100 ms are consistent with the lag of 30 ms, followed by rapid decay, observed in synchrotron footprinting experiments (Gupta *et al.*, 2007 ▸). This lag has been attributed to quenching of ·OH by buffer molecules. In addition, as detailed earlier, solvent composition has also been found to have a significant effect on the rate of cleavage, highlighting the effect of quenching.

These observations, and those illustrating residue sensitivity changing as a function of solvent accessibility, suggest solvent quenching and diffusion delay are significant effects and a probable cause of a dose-rate effect in RT synchrotron millisecond X-ray crystallography. However, it will only be possible to fully establish which processes dominate when advances in detector technology make much shorter timescales accessible. If diffusion and quenching dominate, the observation of an ∼50% reduction in FcγRIIIa lifetime when a pause is introduced during the experiment (Fig. 1 ▸ *a*) can be simply explained by the increased movement of main-chain fragments disrupting the crystal lattice and the diffusion of remaining radicals through the lattice. This slow lattice disruption is likely to be the origin of the radiation-damage dark progression previously observed on the second–minute timescale above 180 K (Warkentin *et al.*, 2011 ▸, 2012 ▸). On shorter timescales, and above ∼160 K when ·OH becomes mobile, radical diffusion is the origin of a dose-rate effect. The increased crystal lifetime observed at 4 s during continuous data collection again points to diffusion and quenching as important effects. During continuous data collection the dose is deposited in the crystal over a longer time period; radical recombination should therefore be greatly reduced, with a concomitant decrease in crystal lifetime. The increase in lifetime observed suggests that in this regime recombination plays a minor role compared with quenching and diffusion. At higher dose rates recombination of radical species will play an increasingly important role, but with the data currently available it is not possible to establish whether this or quenching and diffusion within the solvent is the origin of the dose-rate effect observed in these experiments.

If this model of diffusion, quenching and recombination of radicals resulting in increased lifetimes during continuous data collection and at high dose-rates is correct, it not only explains our results but also suggests that massive gains in crystal lifetime might be achieved with greater flux densities. This would allow many more data to be collected during an initial ‘lag phase’ lasting a few tens of milliseconds while significant radical recombination occurs and before large numbers of radicals can diffuse through the solvent resulting in rapid crystal decay. In the limit, it might be possible to approach the two-orders-of-magnitude increase in crystal lifetime achieved by cryocooling to 100 K. Given the attractions of *in situ* data collection even in the face of the present highly fragmented data-collection regimes (RT data sets for large viruses have typically required many hundreds of crystals; Grimes *et al.*, 1998 ▸; Abrescia *et al.*, 2004 ▸, 2008 ▸), this would be likely to transform the practice of RT macromolecular crystallography.

In the short term, development of data-collection strategies to take advantage of the possibilities of fast-readout PADs combined with a microcalibre ‘machine-gun’ approach to depositing radiation at very high dose rates into small regions of larger crystals might prove effective. The dependence of the RT crystal lifetime on radical quenching and diffusion means that it is also worthwhile exploring the optimization of radical quenching buffers, possibly adding to the current highly successful crystallization screens to build in this added value for RT crystallography. In the medium term, we note that the next generation of cryo- and superconducting undulators promises a significant increase (up to an order of magnitude at 12 keV) in photon flux from the current generation of light sources, whilst the trend towards faster read-out detectors promises to deliver matching increases in performance. In the light of our experiences, described here and in Axford (2012 ▸), we believe that it will also be worthwhile devoting greater effort to optimizing data analysis from the fragmentary data which are likely to be acquired by the increased application of the so-called ‘American Method’ of ‘shoot first and ask questions later’ (Rossmann & Erickson, 1983 ▸). For the cases of data collection for structure determination of BEV 2 and FcγRIIIa (Axford *et al.*, 2012 ▸), data collection and analysis was based on practices established through experience and was effective but very labour-intensive. In the case of the structure solution of two polymorphs of FcγRIIIa (F158V) a total of 260° of data were collected from each crystal type, with the number of crystals required varying between 44 (158F) and 72 (158V) (Axford *et al.*, 2012 ▸). Diffraction data were collected in a suboptimal fashion (given the results in this present work) with an exposure time of 0.1 s. Taking advantage of the dose-rate effect observed in FcγRIIIa (Fig. 2 ▸) would have permitted the same amount of data to be collected from 30 (158F) and 50 (158V) crystals: a significant reduction. Increases in incident flux and detector read-out speed promise further gains. In the meantime such protein and virus data collection is feasible, but major challenges remain in increasing the level of automation and improving the robustness of data-integration programs so that they can cope routinely with thin wedges of very weak fine-sliced data.

## 5. Conclusions   

The data presented here provide clear evidence for a dose-rate effect in RT crystallography when using the full flux of an undulator beamline. The effect becomes significant at exposure times less than 60 ms (frame rates of >16 Hz), with lifetimes increasing as a function of dose rate. Also apparent is a reduced crystal lifetime when there is a pause between frames within a data set when, for example, the X-ray detector is read out. Both of these observations can be explained by considering a three-part model for radiation damage at room temperature. On slow timescales (>1 s) radical diffusion and quenching within the solvent occur, while on fast timescales (<60 ms) radical diffusion, quenching and recombination are relevant. Differences in free-radical formation and propagation at RT, the latter of which is quite different between RT and 100 K, account for the absence of a significant dose-rate effect in cryocrystallography. These observations suggest that more intense beams and faster detectors might render RT data collection a generally attractive strategy for the collection of macromolecular crystallography data.

## Supplementary Material

Supporting information file. DOI: 10.1107/S0907444912012553/tz5006sup1.pdf

## Footnotes

1 Supplementary material has been deposited in the IUCr electronic archive (Reference: TZ5006). Services for accessing this material are described at the back of the journal.

2 For the crystals used in this study, the solvent contents are 63, 83 and 69% for FcγRIIIA, BEV and A_2A_AR, respectively.

## Appendix A Count-rate correction

The Pilatus 6M is a paralyzable detector and as such a dead-time correction must be made at high count rates. The correction is of the form *C* = *C*_0_exp(−*C*_0_τ), where *C*_0_ is the measured count rate, *C* is the corrected count rate and τ is the dead time. For the experiments carried out here the dead time was 199 ns. This correction is applied by the detector software. The combination of the high fluxes and short exposure times used in this study mean that it is important to confirm that the correction made is valid, as if it were not the effect would be to underestimate intense reflections, distorting the intensity decay curves. Supplementary Fig. S1^1^ shows the count-rate correction curve for the I24 Pilatus 6M. The *y* axis is marked at the maximum count rate recommended by the manufacturer and at the maximum count rate observed for each of the systems included in this work. It is clear from the plot that the experiments carried out here are well within the valid range of the dead-time correction model.

## Appendix B UV–Vis of irradiated water

UV–Vis absorption data were also collected from thin films of pure water at I24. Several factors combine to make the collection of low-wavelength (<250 nm) data difficult: the presence of microcrystalline ice makes spectra noisy, the intensity of the deuterium halogen lamp is low in this wavelength region and optical losses become large. Nonetheless, a clear absorbance shoulder was observed to form at the low-wavelength limit (Supplementary Fig. S2^1^) upon exposure to X-­rays, indicating the formation of hydroxyl radicals (Janik *et al.*, 2007 ▸). We believe this to be a shoulder rather than a distinct band centred at 280 nm owing to the instrument and sample limitations outlined above. Such a shoulder is in agreement with previous work on the radiolysis of pure water. Despite these limitations and the relatively weak absorbance of the hydroxyl radical, it is clear that an X-ray-induced change in absorbance is readily observed using the Diamond microspectrophotometer. It should be noted that the presence of cryoprotectants in the solutions studied in Fig. 4 ▸ greatly reduces sample limitations (with respect to UV–Vis absorption spectroscopy), with the result that the low-wavelength limit is lowered.

## References

[bb1] Abrescia, N. G., Cockburn, J. J., Grimes, J. M., Sutton, G. C., Diprose, J. M., Butcher, S. J., Fuller, S. D., San Martín, C., Burnett, R. M., Stuart, D. I., Bamford, D. H. & Bamford, J. K. (2004). *Nature (London)*, **432**, 68–74. 10.1038/nature03056 15525981

[bb2] Abrescia, N. G., Grimes, J. M., Kivelä, H. M., Assenberg, R., Sutton, G. C., Butcher, S. J., Bamford, J. K., Bamford, D. H. & Stuart, D. I. (2008). *Mol. Cell*, **31**, 749–761. 10.1016/j.molcel.2008.06.026 18775333

[bb3] Axford, D. *et al.* (2012). *Acta Cryst.* D**68**, 592–600. 10.1107/S0907444912006749 PMC4791750 22525757

[bb4] Barker, A. I., Southworth-Davies, R. J., Paithankar, K. S., Carmichael, I. & Garman, E. F. (2009). *J. Synchrotron Rad.* **16**, 205–216. 10.1107/S0909049509003343 19240332

[bb5] Bingel-Erlenmeyer, R., Olieric, V., Grimshaw, J. P. A., Gabadinho, J., Wang, X., Ebner, S. G., Isenegger, A., Schneider, R., Schneider, J., Glettig, W., Pradervand, C., Panepucci, E. H., Tomizaki, T., Wang, M. & Schulze-Briese, C. (2011). *Cryst. Growth Des.* **11**, 916–923.

[bb6] Blake, C. & Phillips, D. (1962). *Proceedings of the Symposium on the Biological Effects of Ionising Radiation at the Molecular Level*, pp. 183–191. Vienna: International Atomic Energy Agency.

[bb7] Blundell, S. & Johnson, L. (1976). *Protein Crystallography*. New York: Academic Press.

[bb8] Brivati, J. A., Symons, M. C. R., Tinling, D. J. A. & Williams, D. O. (1969). *J. Chem. Soc. A*, pp. 719–720.

[bb9] Buxton, G. V. (1987). *Radiation Chemistry: Principles and Applications*, edited by Farhataziz & M. A. J. Rodgers, pp. 321–348. New York: VCH.

[bb10] Chapman, H. N. *et al.* (2011). *Nature (London)*, **470**, 73–77.

[bb11] Cherezov, V., Riedl, K. M. & Caffrey, M. (2002). *J. Synchrotron Rad.* **9**, 333–341. 10.1107/s0909049502014528 12409619

[bb12] De la Mora, E., Carmichael, I. & Garman, E. F. (2011). *J. Synchrotron Rad.* **18**, 346–357. 10.1107/S0909049511007163 21525642

[bb13] Dorfman, L. & Adams, G. (1973). *Reactivity of the Hydroxyl Radical in Aqueous Solutions.* Washington: US National Bureau of Standards.

[bb14] Eikenberry, E. F., Brönnimann, C., Hülsen, G., Toyokawa, H., Horisberger, R., Schmitt, B., Schulze-Briese, C. & Tomizaki, T. (2003). *Nucl. Instrum. Methods A*, **501**, 260–266. 10.1107/S0909049505038665 16495612

[bb15] Fraser, J. S., van den Bedem, H., Samelson, A. J., Lang, P. T., Holton, J. M., Echols, N. & Alber, T. (2011). *Proc. Natl Acad. Sci. USA*, **108**, 16247–16252. 10.1073/pnas.1111325108 PMC3182744 21918110

[bb16] Garman, E. F. (2010). *Acta Cryst.* D**66**, 339–351. 10.1107/S0907444910008656 PMC2852297 20382986

[bb17] Garman, E. & Owen, R. L. (2007). *Methods Mol. Biol.* **364**, 1–18. 10.1385/1-59745-266-1:1 17172757

[bb18] Grimes, J. M., Burroughs, J. N., Gouet, P., Diprose, J. M., Malby, R., Ziéntara, S., Mertens, P. P. & Stuart, D. I. (1998). *Nature (London)*, **395**, 470–478. 10.1038/26694 9774103

[bb19] Gupta, S., Sullivan, M., Toomey, J., Kiselar, J. & Chance, M. R. (2007). *J. Synchrotron Rad.* **14**, 233–243. 10.1107/S0909049507013118 17435298

[bb20] Hill, M. A. & Smith, F. A. (1994). *Radiat. Phys. Chem.* **43**, 265–280.

[bb21] Howells, M. R., Beetz, T., Chapman, H. N., Cui, C., Holton, J. M., Jacobsen, C. J., Kirz, J., Lima, E., Marchesini, S., Miao, H., Sayre, D., Shapiro, D. A., Spence, J. C. H. & Starodub, D. (2009). *J. Electron Spectrosc. Relat. Phenom.* **170**, 4–12. 10.1016/j.elspec.2008.10.008 PMC2867487 20463854

[bb22] Hug, G. (1981). *J. Phys. Chem. A*, **111**, 1835–1843.

[bb23] Ivanova, E., Suvorov, O. & Yudin, I. (2009). *High Energ. Chem.* **43**, 263–268.

[bb24] Jacquamet, L., Ohana, J., Joly, J., Borel, F., Pirocchi, M., Charrault, P., Bertoni, A., Israel-Gouy, P., Carpentier, P., Kozielski, F., Blot, D. & Ferrer, J.-L. (2004). *Structure*, **12**, 1219–1225. 10.1016/j.str.2004.04.019 15242598

[bb25] Janik, I., Bartels, D. M. & Jonah, C. D. (2007). *J. Phys. Chem. A*, **111**, 1835–1843. 10.1021/jp065992v 17309240

[bb26] Jones, G. D., Lea, J. S., Symons, M. C. & Taiwo, F. A. (1987). *Nature (London)*, **330**, 772–773. 10.1038/330772a0 2827033

[bb27] Juers, D. H. & Weik, M. (2011). *J. Synchrotron Rad.* **18**, 329–337. 10.1107/S0909049511007631 21525640

[bb28] Klassen, N. (1987). *Radiation Chemistry: Principles and Applications*, edited by Farhataziz & M. A. J. Rodgers, pp. 29–64. New York: VCH.

[bb29] Kmetko, J., Warkentin, M., Englich, U. & Thorne, R. E. (2011). *Acta Cryst.* D**67**, 881–893. 10.1107/S0907444911032835 PMC3176623 21931220

[bb30] Lebon, G., Warne, T., Edwards, P. C., Bennett, K., Langmead, C. J., Leslie, A. G. & Tate, C. G. (2011). *Nature (London)*, **474**, 521–525. 10.1038/nature10136 PMC3146096 21593763

[bb31] Mao, W. L., Mao, H., Goncharov, A. F., Struzhkin, V. V., Guo, Q., Hu, J., Shu, J., Hemley, R. J., Somayazulu, M. & Zhao, Y. (2002). *Science*, **297**, 2247–2249. 10.1126/science.1075394 12351785

[bb32] McGeehan, J., Ravelli, R. B. G., Murray, J. W., Owen, R. L., Cipriani, F., McSweeney, S., Weik, M. & Garman, E. F. (2009). *J. Synchrotron Rad.* **16**, 163–172. 10.1107/S0909049509001629 PMC2651762 19240328

[bb33] Meents, A., Gutmann, S., Wagner, A. & Schulze-Briese, C. (2010). *Proc. Natl Acad. Sci. USA*, **107**, 1094–1099. 10.1073/pnas.0905481107 PMC2798883 20080548

[bb34] Neutze, R., Wouts, R., van der Spoel, D., Weckert, E. & Hajdu, J. (2000). *Nature (London)*, **406**, 752–757. 10.1038/35021099 10963603

[bb35] Owen, R. L., Yorke, B. A., Gowdy, J. A. & Pearson, A. R. (2011). *J. Synchrotron Rad.* **18**, 367–373. 10.1107/S0909049511004250 PMC3083913 21525644

[bb36] Paithankar, K. S. & Garman, E. F. (2010). *Acta Cryst.* D**66**, 381–388. 10.1107/S0907444910006724 PMC2852302 20382991

[bb37] Paithankar, K. S., Owen, R. L. & Garman, E. F. (2009). *J. Synchrotron Rad.* **16**, 152–162. 10.1107/S0909049508040430 19240327

[bb38] Rajendran, C., Dworkowski, F. S. N., Wang, M. & Schulze-Briese, C. (2011). *J. Synchrotron Rad.* **18**, 318–328. 10.1107/S090904951100968X PMC3133521 21525639

[bb39] Rossmann, M. G. & Erickson, J. W. (1983). *J. Appl. Cryst.* **16**, 629–636.

[bb40] Sauter, N. K. & Poon, B. K. (2010). *J. Appl. Cryst.* **43**, 611–616. 10.1107/S0021889810010782 PMC2875182 20502598

[bb41] Sclavi, B., Woodson, S., Sullivan, M., Chance, M. R. & Brenowitz, M. (1997). *J. Mol. Biol.* **266**, 144–159. 10.1006/jmbi.1996.0775 9054977

[bb42] Snell, E. H., Bellamy, H. D., Rosenbaum, G. & van der Woerd, M. J. (2007). *J. Synchrotron Rad.* **14**, 109–115. 10.1107/S090904950604605X 17211077

[bb43] Southworth-Davies, R. J. & Garman, E. F. (2007). *J. Synchrotron Rad.* **14**, 73–83. 10.1107/S0909049506044177 17211073

[bb44] Southworth-Davies, R. J., Medina, M., Carmichel, I. & Garman, E. (2008). *Structure*, **12**, 1531–1541. 10.1016/j.str.2007.10.013 18073104

[bb45] Swartz, H. M. & Swartz, S. M. (1983). *Methods Biochem. Anal.* **29**, 207–323. 10.1002/9780470110492.ch5 6304458

[bb46] Symons, M. (1995). *Radiat. Phys. Chem.* **45**, 837–845.

[bb47] Symons, M. (1999). *Prog. React. Kinet. Mech.* **24**, 139–164.

[bb48] Takamoto, K. & Chance, M. R. (2006). *Annu. Rev. Biophys. Biomol. Struct.* **35**, 251–276. 10.1146/annurev.biophys.35.040405.102050 16689636

[bb49] Walter, T. S. *et al.* (2005). *Acta Cryst.* D**61**, 651–657. 10.1107/S0907444905007808 PMC7159505 15930615

[bb50] Warkentin, M., Badeau, R., Hopkins, J. B., Mulichak, A. M., Keefe, L. J. & Thorne, R. E. (2012). *Acta Cryst.* D**68**, 124–133. 10.1107/S0907444911052085 PMC3266852 22281741

[bb51] Warkentin, M., Badeau, R., Hopkins, J. & Thorne, R. E. (2011). *Acta Cryst.* D**67**, 792–803. 10.1107/S0907444911027600 PMC3169314 21904032

[bb52] Xu, G. & Chance, M. R. (2007). *Chem. Rev.* **107**, 3514–3543. 10.1021/cr0682047 17683160

[bb53] Zakurdaeva, O. A., Nesterov, S. V. & Feldman, V. I. (2005). *High Energ. Chem.* **39**, 201–206.

