# Supplementary material for: Outrunning free radicals in room-temperature macromolecular crystallography
Source: Acta Crystallogr D Biol Crystallogr. 2012 Jun 15;68(Pt 7):810–8. doi: 10.1107/S0907444912012553 (PMC4791751; doi:10.1107/S0907444912012553)
Supplement: Supplementary file 1 [file d-68-00810-sup1.pdf]

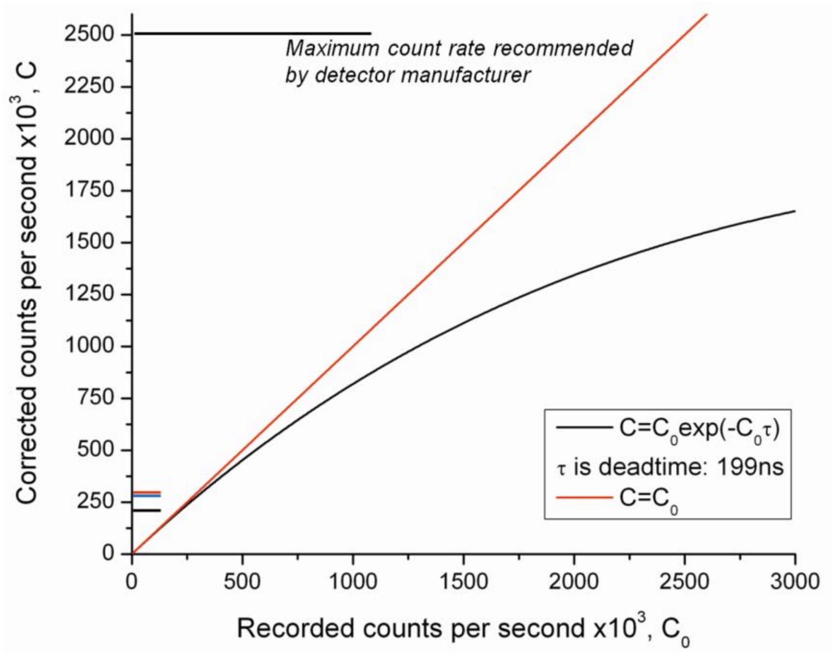

Figure S1 Count rate correction applied to data collected at 25 frames per second. The maximum count-rates observed for FcγRIIIa, BEV and A_{2A}AR crystals are marked on the y-axis as blue, black and red lines respectively. In all cases the correction is small and well within the valid range of the deadtime correction model. The magnitude of the correction made for the most intense reflection observed in this work (from an A_{2A}AR crystal) was 5.6%.

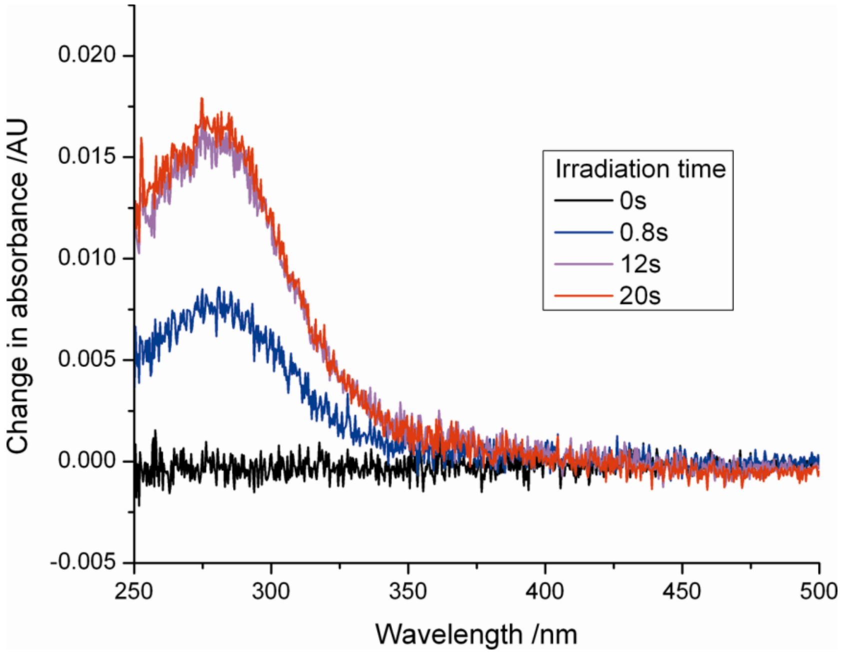

Figure S2 UV-Vis absorption difference spectra of water irradiated by X-rays at I24. A single spectrum collected before X-ray irradiation was used as a reference and subtracted from subsequent spectra. For data collection an exposure time of 0.04s was used with 50 accumulations per spectrum. The black line shows UV-Vis difference spectra before irradiation. The times given for other lines refer to the time UV-Vis data collection was started after the X-ray shutter was opened.
